# Integrin β1 regulates the invasion and radioresistance of laryngeal cancer cells by targeting CD147

**DOI:** 10.1186/s12935-018-0578-z

**Published:** 2018-06-07

**Authors:** Li Li, Xiaoxia Dong, Feng Peng, Li Shen

**Affiliations:** 1 The Functional Science Laboratory, School of Basic Medical Sciences, Hubei University of Medicine, Shiyan, 442000 Hubei People’s Republic of China; 2 Department of Pharmacology, School of Basic Medical Sciences, Hubei University of Medicine, Shiyan, 442000 Hubei People’s Republic of China; 3 Department of Clinical Oncology, Taihe Hospital, Hubei University of Medicine, 30 South Renmin Road, Shiyan, 442000 Hubei People’s Republic of China

**Keywords:** Integrin β1, CD147, Invasion, Radioresistance, Laryngeal cancer

## Abstract

**Background:**

Increased expression of integrin β1 has been reported to correlate with progression and therapy resistance in many types of cancers. The aim of this study was to investigate the effects of integrin β1 on the invasion and radioresistance of laryngeal cancer cells.

**Methods:**

The expression of integrin β1 in the tumor specimens of laryngeal cancer patients was assessed by immunohistochemical assays. The invasion ability of laryngeal cancer cells was detected by transwell and wound healing assays. The radiosensitivity of laryngeal cancer cells was evaluated by flow cytometry and colony formation assays.

**Results:**

High expression of integrin β1 was significantly associated with lymph node metastasis, TNM stage and poor clinical outcomes (all *p *< 0.05). Knockdown of integrin β1 in laryngeal cancer cells inhibited invasion and increased radiosensitivity. Mechanistically, these effects were caused by suppression of the downstream focal adhesion kinase (FAK)/cortactin pathway. In addition, integrin β1 could interact with CD147 and the antibody blockade of CD147 led to the deactivation of FAK/cortactin signaling. Further studies revealed that the interaction between integrin β1 and CD147 relied on intact lipid rafts. Disruption of lipid rafts by methyl beta cyclodextrin in laryngeal cancer cells was able to reverse integrin β1-mediated malignant phenotypes.

**Conclusions:**

Integrin β1 has potential as a therapeutic target in prevention and treatment of laryngeal cancer.

## Background

Laryngeal cancer is a common respiratory cancer and is one of the leading causes of morbidity and mortality worldwide, especially in China [1, 2]. Because of limited therapeutic modalities, the prognosis of patients with laryngeal cancer after curative treatment remains unsatisfactory [3]. The most common treatment options for laryngeal cancer are radiation therapy, surgery and chemotherapy. But patients often experience disease relapse due to eventual tumor metastasis and emergence of therapy resistance [4]. Thus, there is an urgent need to identify new target molecules for improving treatment and overcoming therapy resistance of laryngeal cancer.

Integrins, a family of transmembrane cell surface receptors, are composed of 18 α and 8 β subunits [5]. Integrins activate various signaling pathways, which contribute to the regulation of cell proliferation, migration, invasion, and therapy resistance [6, 7]. Integrin β1 is a critical regulator of cancer initiation and progression. In addition, integrin β1 has been linked to therapeutic resistance including conventional radiotherapy and chemotherapy in various tumor entities [8–11]. Aberrant integrin signaling has been implicated in laryngeal cancer metastasis [12]. However, the underlying mechanisms of how the increased expression of integrin β1 confers invasion and radioresistance of laryngeal cancer remain unclear.

In this study, we first measured the expression of integrin β1 in the tumor specimens of laryngeal cancer patients. Next, we investigated the biological function of integrin β1 in modulating the invasion and radioresistance of laryngeal cancer cells. Lipid rafts are detergent-insoluble, cholesterol-rich microdomains of the plasma membrane [13]. It is generally accepted that the localization of integrin β1 is lipid rafts-dependent [14]. Here, we also evaluated the effects of lipid rafts on integrin β1-mediated malignant phenotypes. Our findings suggest a promising approach to treat laryngeal cancer by targeting integrin β1.

## Methods

### Clinical samples and immunohistochemistry

From 2010 to 2016, 60 laryngeal cancer tissues and 25 noncancerous laryngeal tissues were obtained from patients who underwent surgery at Taihe hospital, Hubei University of Medicine. The tissues were incubated with integrin β1 antibody (1:300; Abcam, Cambridge, MA, USA) at 4 °C overnight. After washing in PBS, the tissues were incubated with HRP-labeled secondary antibody (Beyotime, Jiangsu, China) for 30 min at room temperature. Finally, the specific immunostaining was visualized using an ultrasensitive streptavidin-peroxidase system (Maxim Biotech, Fuzhou, China) [15]. The intensity of integrin β1 staining in individual cases was evaluated by two independent scorers in a blinded fashion. A score > 4 was defined as high expression, and a score ≤ 4 was regarded as low expression [16].

### Cell culture and transfection

Human laryngeal cancer cell lines (Hep-2, TU686 and M4e) were purchased from the Cell Bank of Chinese Academy of Sciences (Shanghai, China). All the cells lines were cultured in DMEM medium (Gibco-BRL, Carlsbad, CA, USA) supplemented with 10% fetal bovine serum (FBS) at 37 °C in 5% CO_2_. One day before transfection, the Hep-2 cells were seeded at a density of 3 × 10^5^ in 6-well culture plates. Then Hep-2 cells were transfected with 20 nmol of integrin β1 siRNA or negative control siRNA (NC) using Lipofectamine 2000 (Invitrogen, Carlsbad, CA, USA) according to the manufacturer’s instruction. The siRNA oligonucleotides were designed and chemically synthesized by GenePharma (Shanghai, China) as shown in Table 1.

**Table 1:** Sequences of integrin β1 siRNA and negative control siRNA

Name	Sequences (5′-3′)
Negative control	
Sense	UUCUCCGAACGUGUCACGUTT
Antisense	ACGUGACACGUUCGGAGAATT
siRNA-1 (integrin β1-472)	
Sense	GGCUCCAAAGAUAUAAAGATT
Antisense	UCUUUAUAUCUUUGGAGCCTT
siRNA-2 (integrin β1-2172)	
Sense	GCCUUCAAUAAAGGAGAAATT
Antisense	UUUCUCCUUUAUUGAAGGCTT
siRNA-3 (integrin β1-2504)	
Sense	GGAGUUUGCUAAAUUUGAATT
Antisense	UUCAAAUUUAGCAAACUCCTT

### Quantitative real-time PCR (qPCR) and western blotting

Total RNA was extracted using Trizol reagent (Invitrogen). And 1 µg of total RNA was reverse-transcribed into cDNA using a M-MLV RT kit (Takara, Dalian, China). QPCR was performed using the SYBR-Green Real-Time PCR Master Mix kit (Toyobo, Osaka, Japan). Primer sequences were as follows: integrin β1 (forward: 5′-GACGCCGCGCGGAAAAGATG-3′, reverse: 5′-GCACCACCCACAATTTGGCCC-3′) and GAPDH (forward: 5′-CCAACCGCGAGAAGATGA-3′, reverse: 5′-CCA GAGGCGTACAGGGATAG-3′). The relative mRNA expression of target genes was identified using 2^−ΔΔCt^ methods. Western blotting was performed as described previously [16]. In brief, 10 µg of total protein was isolated by 10% SDS-PAGE and transferred onto a PVDF membrane. After blocking with 5% non-fat dry milk, the membrane was incubated with primary antibodies. The following antibodies were purchased from Abcam and used in this study: integrin β1 (1:1000), CD147 (1:800), FAK (1:1000), Cortactin (1:1000), pFAK Y397 (1:1000), pCortactin Y421 (1:800), and GADPH (1:2000). The immunoreactive bands were detected using ECL-Plus chemiluminescence (Beyotime). GAPDH served as an internal control in the experiments.

### Wound healing and transwell assays

For the wound healing migration assay, cells were plated in 24-well plates and allowed to attach overnight. Confluent monolayer cells were scraped using 10 µl pipette tips. At the indicated time points (0 and 48 h), the wound areas were photographed under a microscope (Olympus, Tokyo, Japan). For the transwell invasion assay, 1 × 10^5^ cells were added into the upper chamber of an insert precoated with matrigel (Costar, Cambridge, MA, USA). And 100 μl medium containing 20% FBS were added to the lower part of the chamber. After 24 h of incubation, the invaded cells were fixed with methanol and stained with eosin solution (Beyotime).

### Radiosensitivity analysis

The radiosensitivity of cells was determined by flow cytometry and colony formation assays. For cell cycle analysis, 1 × 10^6^ cells treated with or without 4 Gy irradiation using an X-ray machine (X-RAD 320, Precision X-ray) were collected and stained with propidium iodide (PI; Beyotime). For cell apoptosis analysis, 1 × 10^6^ cells treated with or without 4 Gy irradiation were stained with Annexin V-FITC (Beyotime) and PI according to the manufacturer’s protocol. The cell cycle distribution and apoptosis rate were measured using flow cytometry (Becton–Dickinson, Mountain View, CA, USA). For colony formation assay, cells were seeded in increasing numbers (200–6000 cells per 6 cm petri dish) before being irradiated. After 14 days, cells were fixed and stained for colony counting (colonies ≥ 50 cells). The surviving fractions were calculated and irradiations were performed as published previously [17].

### Immunofluorescence

Cells were incubated with or without MβCD (Sigma, St. Louis, MO, USA) for 1 h. Then cells were fixed with 5% formaldehyde, permeabilized with 0.2% Triton X-100, and blocked with 3% BSA. The cells were stained with integrin β1 antibody for 1 h, followed by incubation with Cy3-conjugated secondary antibody (Sigma) for 45 min. For lipid raft marker ganglioside GM1 labeling, cells were incubated with FITC-conjugated cholera toxin subunit B (CTXB, Sigma) for 1 h. Cell nuclei were counterstained with DAPI (Sigma). The colocalization between GM1 and integrin β1 was analyzed using the Olympus confocal software.

### Isolation of lipid rafts

Lipid raft fractions were isolated using the Raft Isolation Kit (Sigma) following the manufacturer’s protocol [18]. Twelve fractions were collected and subjected to western blotting. Fractions 1–4 were marked as raft fractions and fractions 5–12 were labeled as non-raft fractions [19].

### Co-immunoprecipitation (IP)

Co-IP was performed as described previously [20]. Briefly, cell lysates were incubated with the anti-integrin β1 or anti-CD147 antibody overnight at 4 °C. The antibody-protein conjugates were then incubated with protein A/G agarose beads (Thermo Fisher, Rockford, IL, USA) for 4 h at 4 °C. The reaction mixture was washed three times and boiled for 5 min at 100 °C. The samples were run on a 10% SDS-PAGE gel and blotted with the special antibody.

### Statistical analysis

All experiments were performed in triplicate. All statistical analyses were carried out using SPSS 14.0 (SPSS Inc, Chicago, IL). A *p* value of < 0.05 was considered significant. All results were expressed as the mean ± SD. Statistical differences were calculated by Student’s *t* test or Chi square test.

## Results

### High expression of integrin β1 in laryngeal cancer patients is associated with TNM stage, lymph node, and poor survival

**Fig. 1:**
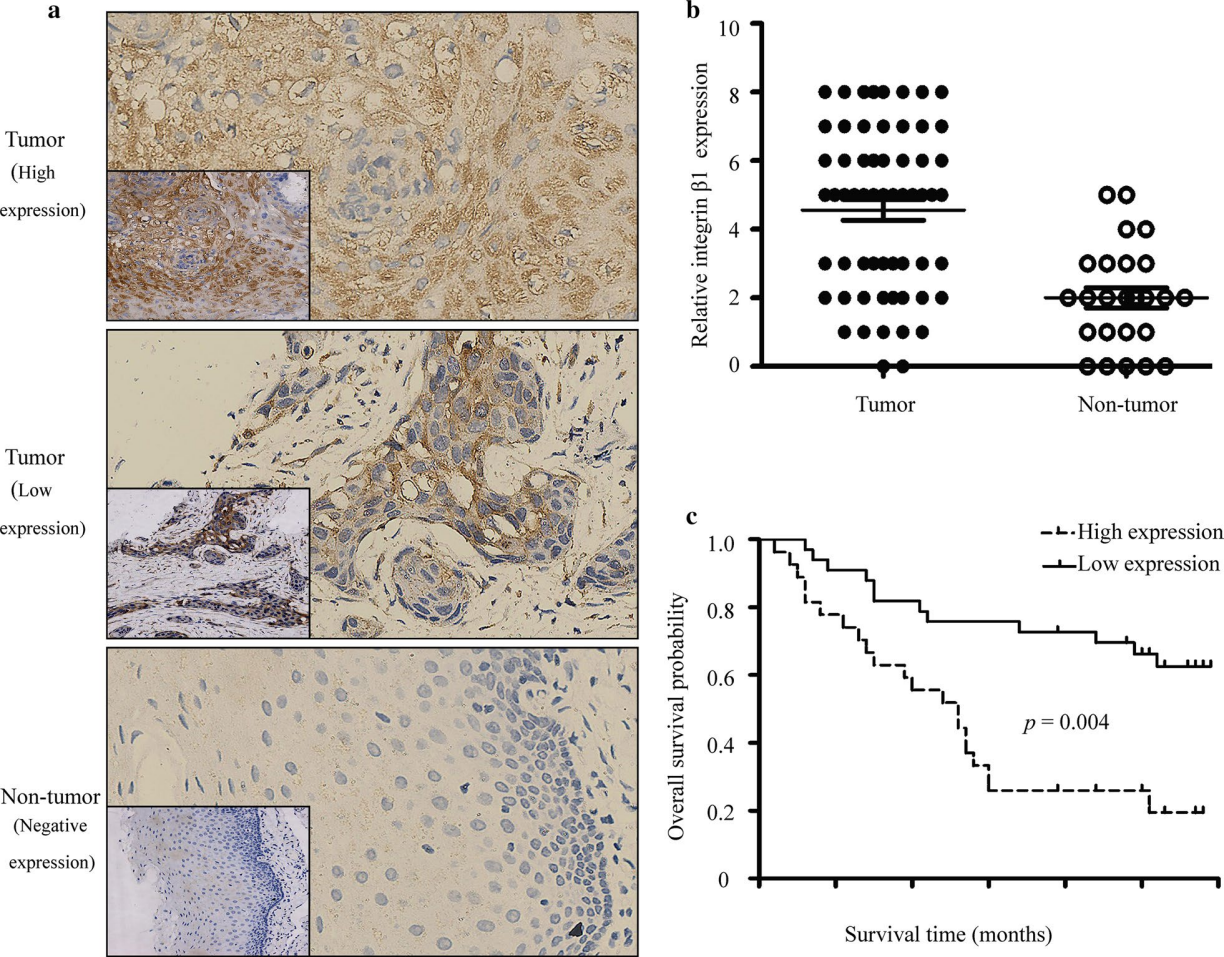
Expression of integrin β1 in laryngeal cancer tissues. **a** Characterization of integrin β1 expression by immunohistochemistry staining. (original magnification ×400, the down-left inserted picture ×100). **b** Analysis of integrin β1 expression based on the immunohistochemistry score. **c** Kaplan–Meier analysis of overall survival for 60 laryngeal cancer patients, grouped according to the expression of integrin β1

Immunohistochemistry was carried out to investigate the expression of integrin β1 in 60 laryngeal cancer tissues and 25 noncancerous laryngeal tissues. As shown in Fig. 1a, integrin β1 was mainly located in the membrane and cytoplasm of most cancer cells. We found that 24 patients possessed low integrin β1 expression while the others had high integrin β1 expression. Integrin β1 protein expression was markedly upregulated in laryngeal cancer tissues in comparison with the expression in non-tumor laryngeal tissues (Fig. 1b). We next assessed the relationship between integrin β1 expression and clinicopathological parameters (Table 2). High expression of integrin β1 was positively correlated with TNM stage (*p* = 0.019) and lymph node metastasis (*p *= 0.005). However, there was no significant correlation between integrin β1 expression and sex, age, tumor diameter or grade of differentiation. Furthermore, a Kaplan–Meier survival analysis showed that the survival rate of laryngeal cancer patients with high integrin β1 expression was significantly lower than that of patients with low integrin β1 expression (*p* = 0.004) (Fig. 1c). These results indicated that integrin β1 may function as a proto-oncogene.

**Table 2:** Relationship between integrin β1 expression and clinicopathological parameters

Clinicopathological parameters	No. of patients	Integrin β1 expression		p value
		Low (n = 24)	High (n = 36)	
Age				
< 60	22	10	12	0.433
≥ 60	38	14	24	
Sex				
Male	44	21	23	0.172
Female	16	3	13	
Tumor diameter				
< 3 cm	25	9	16	0.265
≥ 3 cm	35	15	20	
Grade of differentiation				
Moderate–high	37	18	19	0.349
Low	23	6	17	
Lymph node metastasis				
Positive	47	15	32	0.005^a^
Negative	13	9	4	
TNM stage				
I + II	20	14	6	0.019^a^
III + IV	40	10	30	

^a^ Results of the analysis have statistical significance

### Integrin β1 plays a functional role in laryngeal cancer cell invasion and radioresistance

**Fig. 2:**
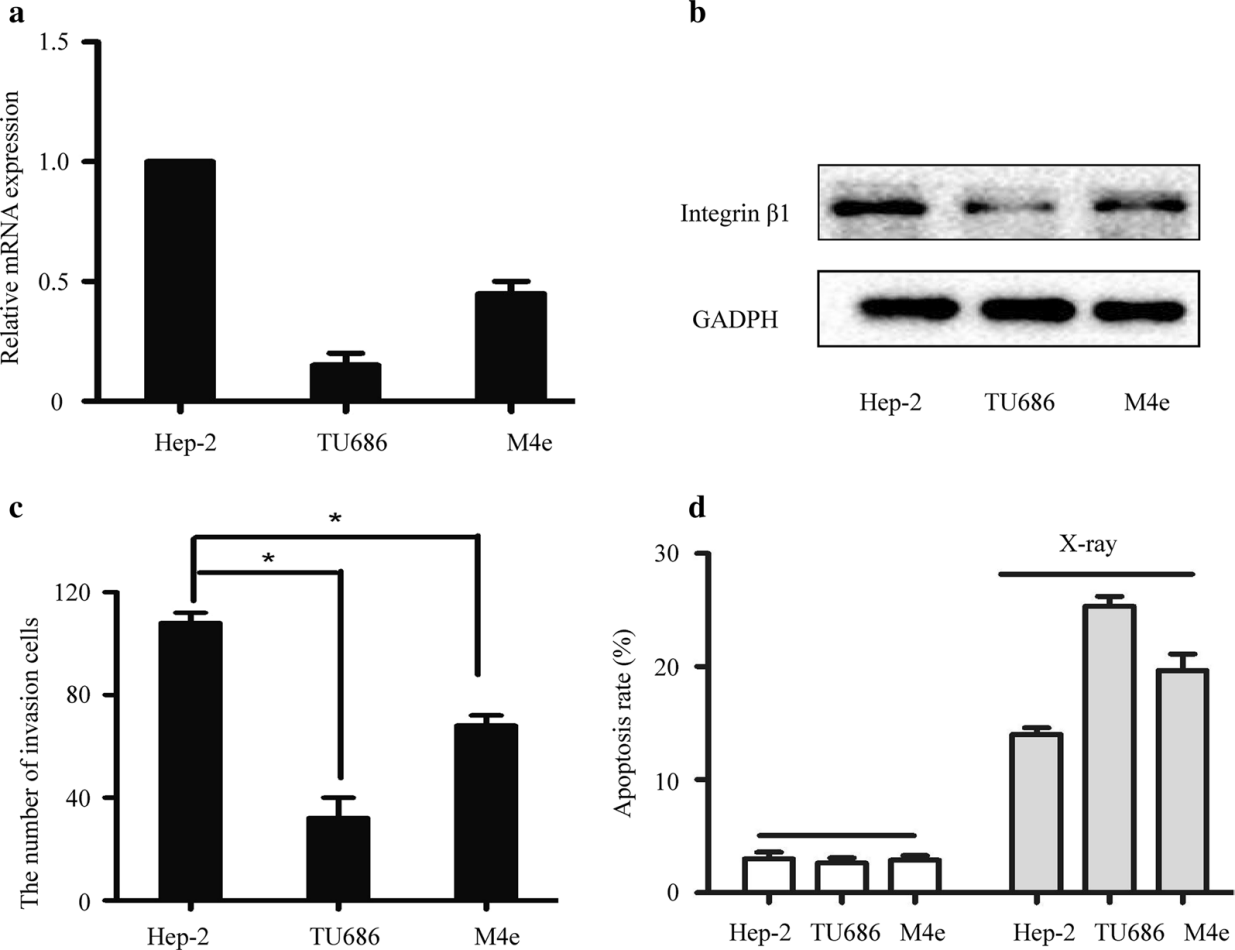
Integrin β1 expression is associated with the invasion and radioresistance of laryngeal cancer cells. **a** The mRNA expression of integrin β1 was analyzed by qPCR. **b** The protein expression of integrin β1 was analyzed by western blotting. GAPDH was used as an internal control. **c** The invasive ability was analyzed by transwell assay. **d** The cell apoptosis was analyzed by flow cytometry. *p < 0.05 compared with Hep-2 cells

The expression of integrin β1 in laryngeal cancer cell lines (Hep-2, TU686 and M4e) was assessed by qPCR and western blotting. Among these cell lines, Hep-2 displayed the highest, whereas TU686 displayed the lowest, levels of integrin β1 mRNA and protein (Fig. 2a and b). Transwell assays showed that the invasive ability of Hep-2 cell lines was stronger than that of other two cell lines (Fig. 2c). Subsequently, all cell lines were treated with 4 Gy irradiation and the results suggested that the apoptosis rate of Hep-2 cells was lower than that of TU686 and M4e cells (Fig. 2d). These results indicated that high expression of integrin β1 was associated with the invasion and radioresistance of laryngeal cancer cells. Thus, Hep-2 cell line was employed in the following studies.

### Inhibition of integrin β1 reduces invasiveness of laryngeal cancer cells

**Fig. 3:**
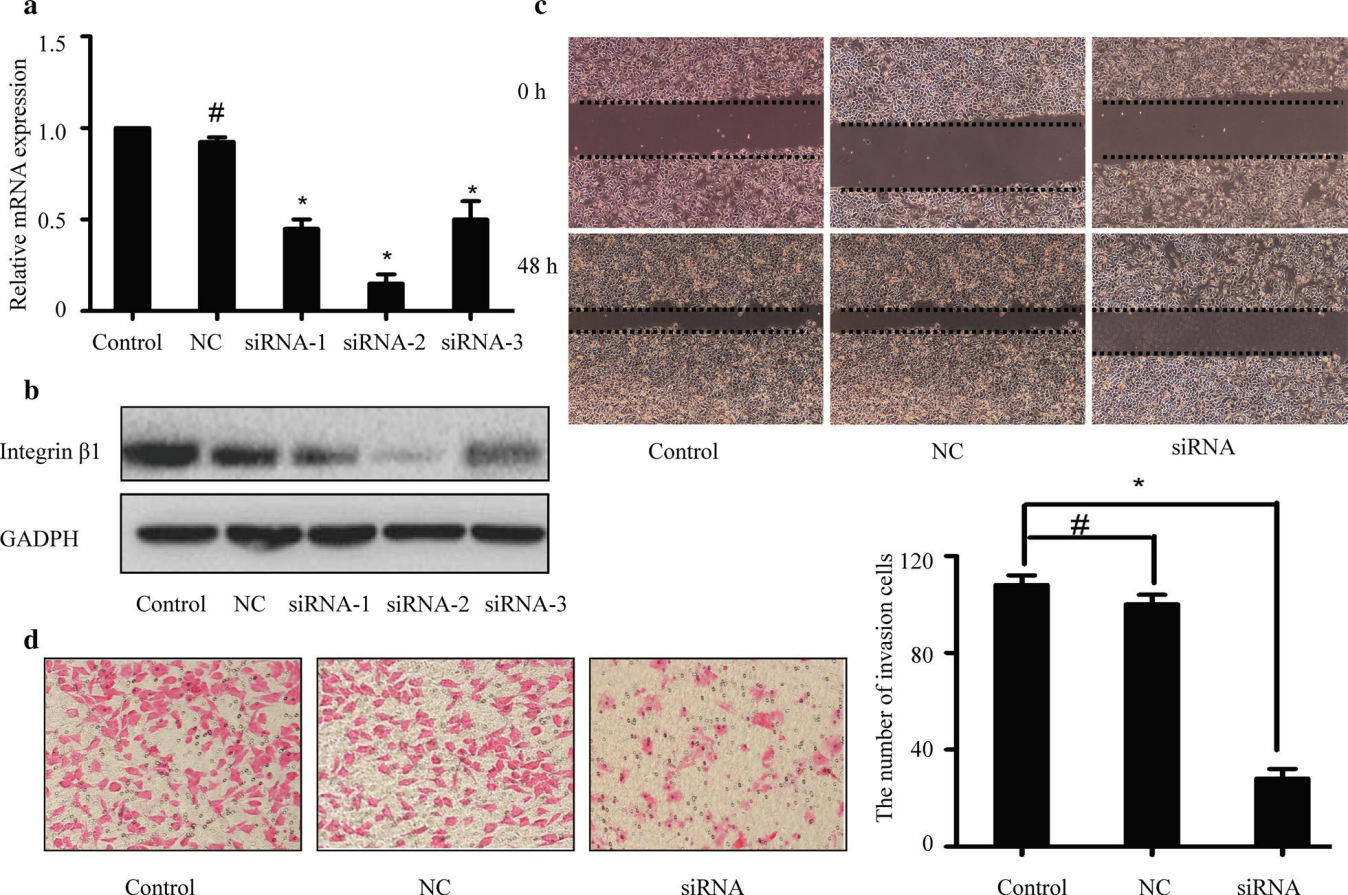
Effects of integrin β1 inhibition on the invasiveness of laryngeal cancer Hep-2 cells. To knockdown the expression of integrin β1, three double-stranded siRNAs targeting integrin β1 or negative control siRNA (NC) were transfected into Hep-2 cells. **a** The mRNA expression of integrin β1 was analyzed by qPCR. **b** The protein expression of integrin β1 was analyzed by western blotting. GAPDH was used as an internal control. The siRNA-2 exhibited the most obvious inhibitory effects and was employed in the following experiments. **c** The migratory ability was analyzed by wound healing assay. **d** The invasive ability was analyzed by transwell assay. *p < 0.05; ^#^*p* > 0.05 compared with each control Hep-2 cells

To knockdown the expression of integrin β1, three double-stranded siRNAs targeting coding regions of integrin β1 gene were synthesized and transfected into Hep-2 cells. Results of qPCR and western blotting showed that the expression of integrin β1 at both the mRNA and protein levels was suppressed (Fig. 3a and b). We also found that siRNA-2 exhibited the most obvious inhibitory effects. Therefore, siRNA-2 was used for the following experiments. Next, we analyzed the effects of integrin β1 inhibition on the migration and invasive abilities of Hep-2 cells by wound healing and transwell assays. As shown in Fig. 3c and d, down-regulation of integrin β1 by siRNA could apparently reduce the migration and invasive abilities of Hep-2 cells (*p *< 0.05).

### Inhibition of integrin β1 increases radiosensitivity of laryngeal cancer cells

**Fig. 4:**
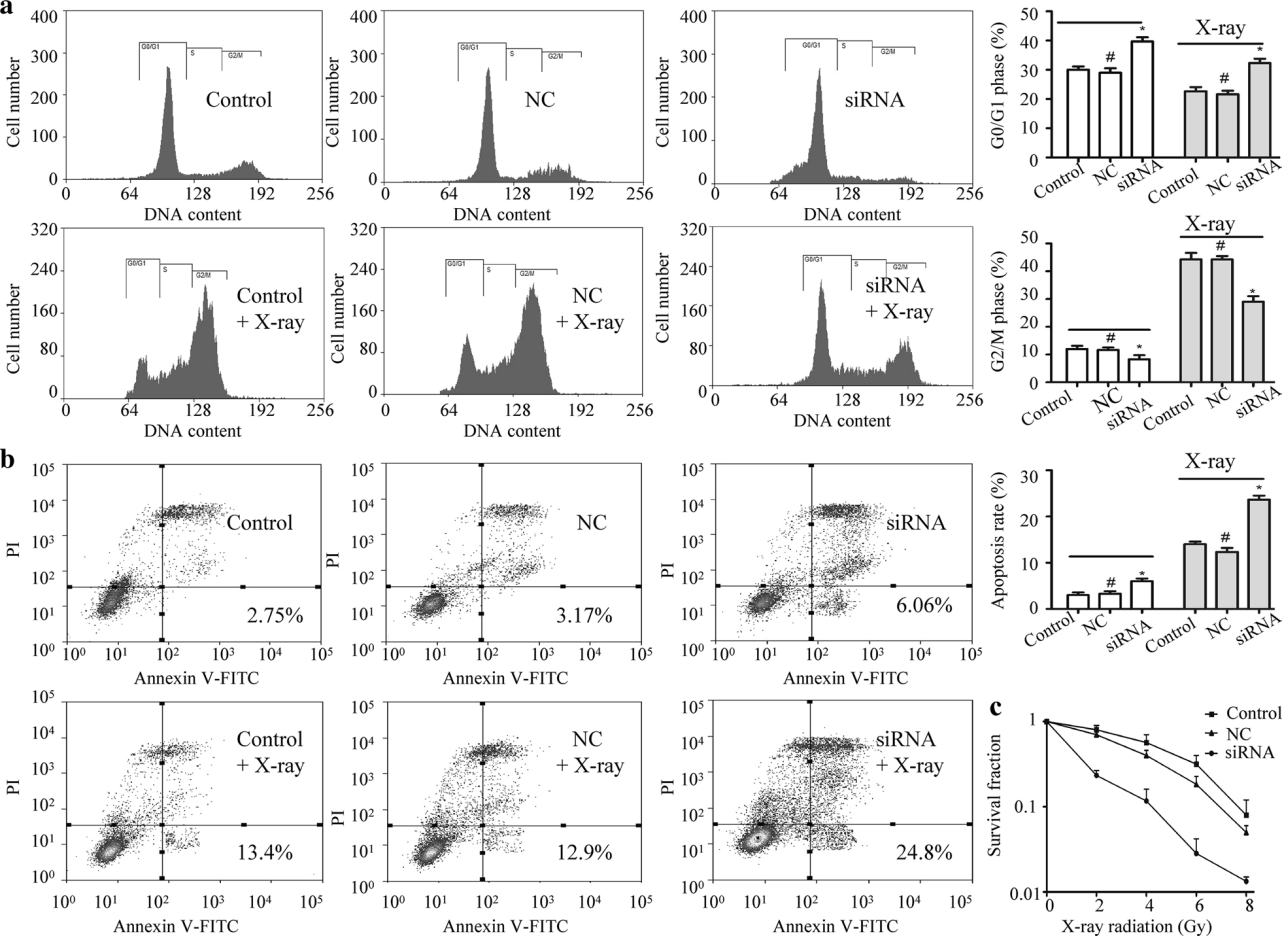
Effects of integrin β1 inhibition on the radioresistance of laryngeal cancer Hep-2 cells. Integrin β1 was knocked down with negative control siRNA (NC) or integrin β1-specific siRNA in Hep-2 cells. **a** The cell cycle distribution was analyzed by flow cytometry. **b** The cell apoptosis was analyzed by flow cytometry. **c** The survival fraction value was analyzed by colony formation assay. *p < 0.05; ^#^*p* > 0.05 compared with each control Hep-2 cells

To determine whether integrin β1 affected radioresistance, integrin β1 was knocked down by siRNA-2 in Hep-2 cells. The cell cycle distribution and apoptosis were analyzed by flow cytometry. As shown in Fig. 4a, the percentage of Hep-2 cells arrested at G2/M phase markly increased after 4 Gy irradiation. However, integrin β1-knockdown cells treated with the same dose of irradiation presented a reduced percentage of cells in G2/M phase. In addition, siRNA-mediated knockdown of integrin β1 in Hep-2 cells increased the rates of apoptosis after exposure to 4 Gy radiation (Fig. 4b). Consistent with this, the results of colony formation assays revealed that integrin β1 siRNA transfected cells showed fewer survival fractions when exposed to various doses of irradiation (Fig. 4c).

### Integrin β1 interacts with CD147 and regulates FAK/cortactin signaling

**Fig. 5:**
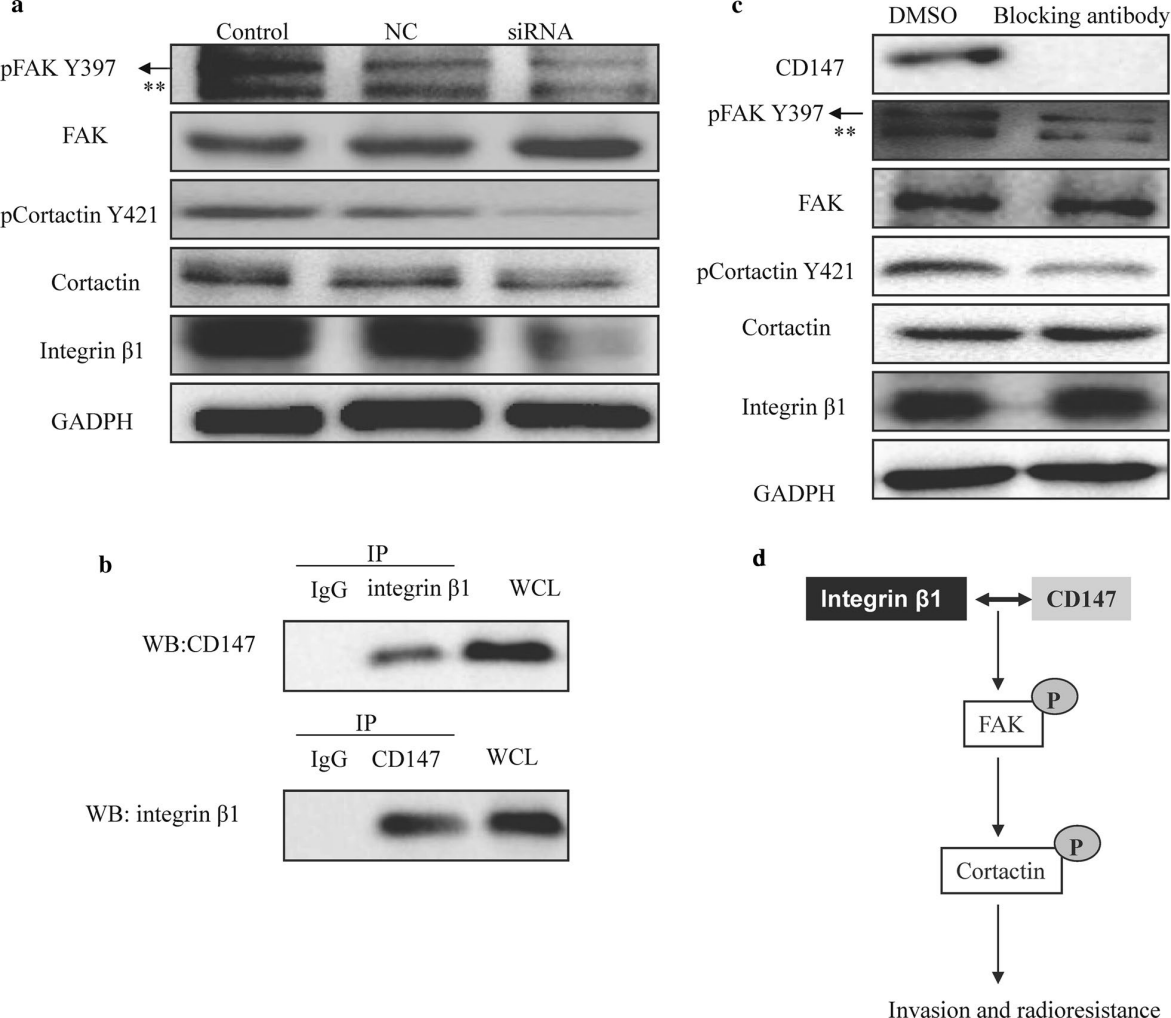
Integrin β1 interacts with CD147 and regulates FAK/cortactin signaling. **a** Effects of integrin β1 inhibition on the expression of FAK/cortactin signaling were analyzed by western blotting. **b** Co-immunoprecipitation of integrin β1 and CD147 in Hep-2 cells. **c** Effects of CD147 blocking antibody on the expression of FAK/cortactin signaling were analyzed by western blotting. **d** Schematic representation of the proposed molecular mechanism of integrin β1. **Unspecific band

Figure 5a showed that knockdown of integrin β1 in Hep-2 cells caused a dephosphorylation of FAK and cortactin. It has been reported that CD147 could interact with integrin β1 in hepatocellular carcinoma [21]. To validate the interaction between CD147 and integrin β1 in laryngeal cancer cells, we performed Co-IP assays. As shown in Fig. 5b, integrin β1 was co-precipitated with CD147; CD147 was pulled down by anti-integrin β1 antibody in Hep-2 cells. Pre-treatment with CD147 blocking antibody inhibited the phosphorylation of FAK and cortactin (Fig. 5c). These findings suggested that integrin β1 regulated the malignant phenotypes of laryngeal cancer cells by interacting with CD147 and activating the downstream FAK/cortactin pathway (Fig. 5d).

### The interaction between integrin β1 and CD147 relies on intact lipid rafts

**Fig. 6:**
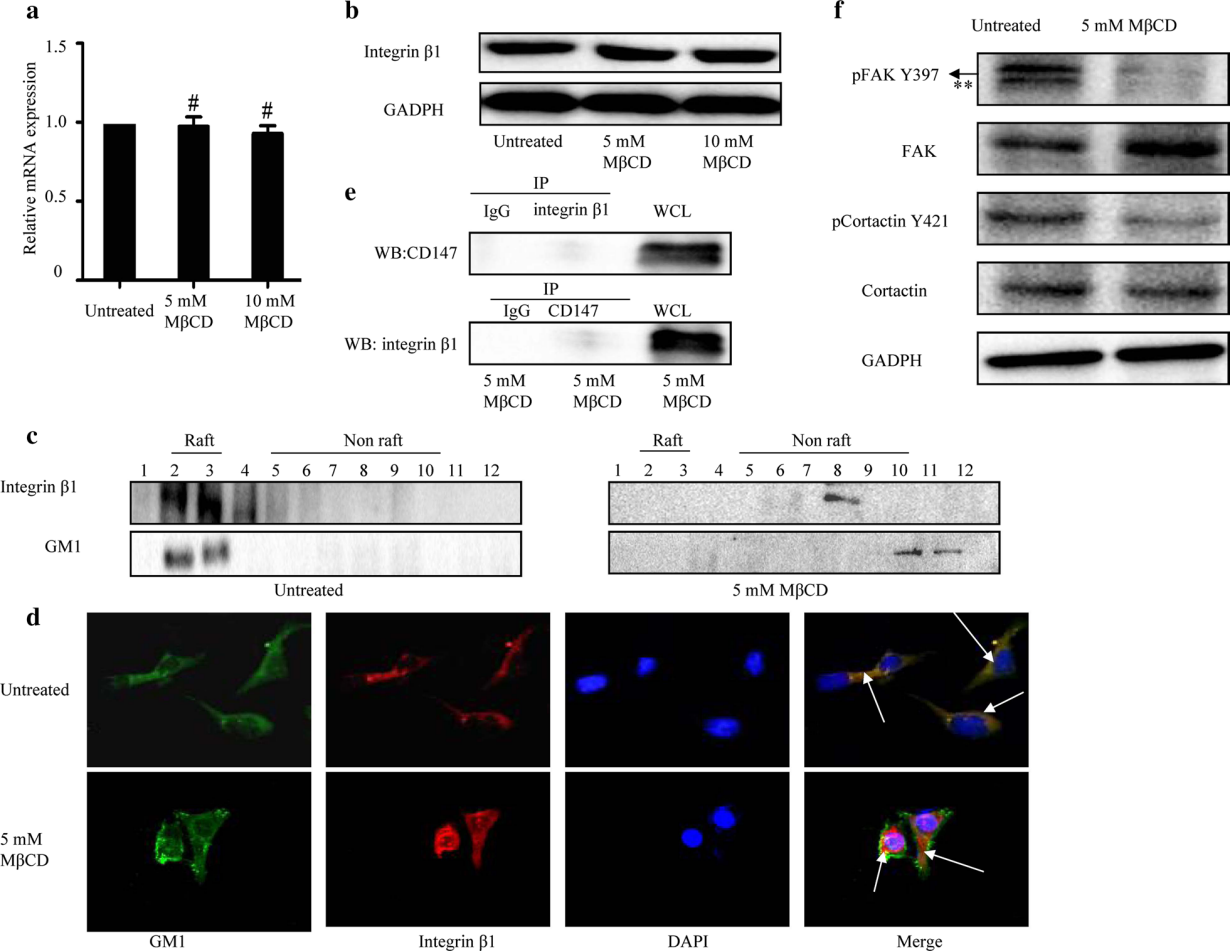
Effects of MβCD on the expression and location of integrin β1 in laryngeal cancer Hep-2 cells. **a** The mRNA expression of integrin β1 was analyzed by qPCR. **b** The protein expression of integrin β1 was analyzed by western blotting. GAPDH was used as an internal control. **c** Location of integrin β1 in lipid raft fractions. **d** Cells treated with MβCD were double-stained for GM1 (green) and integrin β1(red). Cell nuclei were stained with DAPI. **e** Co-immunoprecipitation of integrin β1 and CD147 in Hep-2 cells. **f** The expression of FAK/cortactin signaling was analyzed by western blotting. ^#^*p *> 0.05 compared with untreated Hep-2 cells. ** Unspecific band

The location of integrin β1 in lipid rafts is essential for its function in regulation of cellular events. Thus, we investigated the influence of lipid rafts on the expression of integrin β1 in Hep-2 cells. As shown in Fig. 6a and b, disruption of lipid rafts by MβCD (5 and 10 mM) could not alter the mRNA and protein expression of integrin β1. We then isolated lipid rafts and explored the effects of MβCD on the integrity of lipid rafts. As shown in Fig. 6c, 5 mM MβCD treatment resulted in the relocation of GM1 (a lipid raft marker) to non-raft fractions from lipid raft fractions. We also found that 5 mM MβCD treatment resulted in the redistribution of integrin β1 from lipid raft fractions to non-lipid raft fractions. Additionally, integrin β1 was colocalized with the GM1 in Hep-2 cells. After lipid rafts disruption by MβCD, the colocalization nearly disappeared (Fig. 6d). Figure 6e revealed that MβCD treatment (5 mM) interrupted the interaction between integrin β1 and CD147. MβCD treatment also led to the deactivation of FAK/cortactin signaling (Fig. 6f).

### Disruption of lipid rafts inhibits the invasion and increases the radiosensitivity of laryngeal cancer cells

**Fig. 7:**
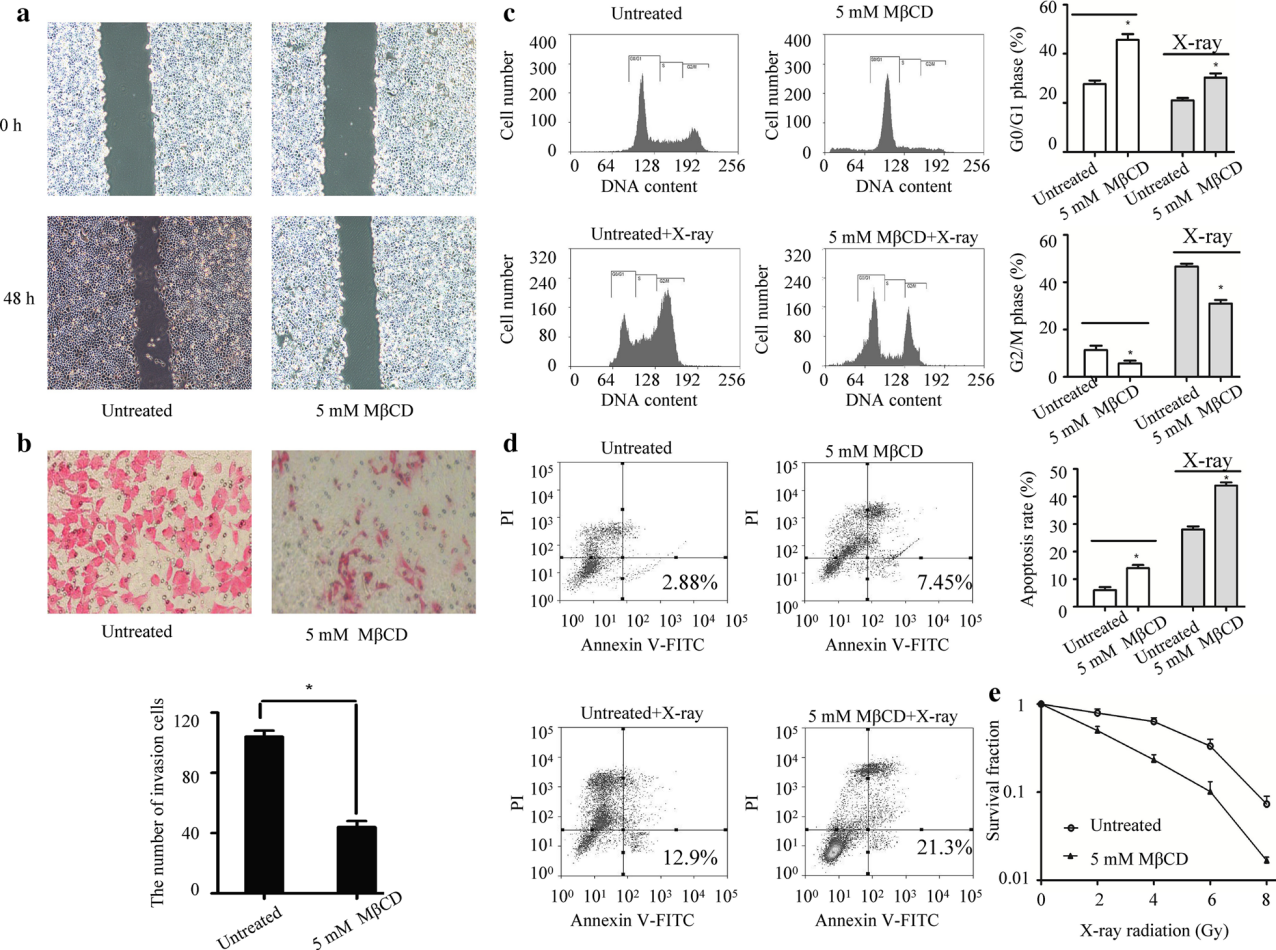
Effects of MβCD on the invasiveness and radioresistance of laryngeal cancer Hep-2 cells. **a** The migratory ability was analyzed by wound healing assay. **b** The invasive ability was analyzed by transwell assay. **c** The cell cycle distribution was analyzed by flow cytometry. **d** The cell apoptosis was analyzed by flow cytometry. **e** The survival fraction value was analyzed by colony formation assay. *p < 0.05 compared with untreated Hep-2 cells

The results from transwell, wound healing, flow cytometry and colony formation experiments showed that disruption of lipid rafts by MβCD reduced invasion and enhanced radiosensitivity of Hep-2 cells (Fig. 7a–e). These findings indicated that the localization of integrin β1 in intact lipid rafts was critical for the invasion and radioresistance of laryngeal cancer cells.

## Discussion

Laryngeal cancer is a highly aggressive malignant tumor with increasing incidence and poor prognosis. And, the emergence of resistance to therapy is a major obstacle in the treatment of laryngeal cancer patients. Exploring relevant factors related to tumorigenesis and development is urgently needed for laryngeal cancer treatment. In the present study, we found that integrin β1 was frequently overexpressed in clinical laryngeal cancer samples. Moreover, integrin β1 expression was correlated with various clinicopathological features including TNM stage and lymph node metastasis. Most importantly, high expression of integrin β1 in laryngeal cancer tissues was associated with poor survival. In addition, loss of function experiments showed that integrin β1 could regulate the invasion and radiosensitivity of laryngeal cancer cells. These results identified integrin β1 as a potential therapeutic target for laryngeal cancer.

Evidence has long accumulated to point toward a key role for integrin β1 in the control of cancer invasion. For example, integrin β1 was a critical effector in promoting the metastasis of esophageal squamous cell carcinoma [22]. Regulation of integrin β1 by miR-199a-5p was involved in breast cancer invasion [23]. Integrin β1 silencing could suppress COX-2-mediated cancer cell invasion in non-small-cell lung cancer [24]. Similarly, our present study confirmed that high expression of integrin β1 was associated with laryngeal cancer invasion. We employed siRNA technology to suppress the expression of integrin β1 in laryngeal cancer cells. The in vitro assays showed that integrin β1 depletion reduced the invasiveness of laryngeal cancer cells.

To date, extensive studies have been performed to investigate the mechanisms of radioresistance in laryngeal cancer. And, a number of molecules have been suggested to be involved in the development of radioresistance. For instance, simultaneous inhibition of HIF-1α and GLUT-1 expression was able to increase the radiosensitivity of laryngeal cancer [25]. MiR-503 might decrease the radioresistance of laryngeal cancer cells via the inhibition of WEE1 [26]. Expression of hPOT1 was reported to be correlated with telomere length and radiosensitivity of laryngeal cancer cells [27]. ALDH1 was shown to act as a predictor of radioresistance in laryngeal cancer [28]. In this study, we demonstrated the role of integrin β1 in mediating the radiosensitivity of laryngeal cancer cells. It is well known that radiation caused apoptosis and induced G2/M cell cycle checkpoint arrest [10, 17]. Our study also revealed that the inhibition of integrin β1 significantly sensitized laryngeal cancer cells to radiation, induced cell apoptosis and reduced G2/M arrest after radiation.

Studies have shown that FAK/cortactin signaling plays important roles in integrin β1-mediated malignant phenotypes. For instance, integrin β1/FAK/cortactin signaling was essential for human head and neck cancer resistance to radiotherapy [7]. Integrin β1/FAK signaling was associated with the invasion and migration of medulloblastoma [29]. Our study confirmed that FAK/cortactin pathways could be suppressed by integrin β1-knockdown, implying their participation in the invasion and radioresistance of laryngeal cancer cells. We also demonstrated that the interaction of integrin β1 with CD147 could activate the downstream FAK/cortactin signaling pathway, subsequently enhancing the malignant properties of laryngeal cancer cells.

As a hallmark of tumor cells, metabolic alterations play a critical role in tumor development [30]. Metabolic reprogramming is also required for both malignant transformation and tumor development, including invasion and metastasis [31]. CD147 is ubiquitously expressed with the highest levels on metabolically active tumor cells and is able to form complex with three major types of transporters (CD98 heavy chain (CD98hc)-L-type amino acid transporter, ASCT2, and monocarboxylate transporters) as well as epithelial cell adhesion molecule (EpCAM) [32]. As an interaction partner of CD147, EpCAM influences the microenvironment within tumors, especially the nutrient microenvironment [33]. Considering that CD147 and its interaction proteins are essential for cellular metabolism, further experiments will be needed to reveal the underlying effects of integrin–CD147 complex and altered metabolism on invasion and radiosensitivity of laryngeal cancer cells.

Lipid rafts have been implicated in cancer cell apoptosis, adhesion and invasion [18, 34]. However, few studies have addressed the effects of lipid rafts on the invasion and radioresistance of laryngeal cancer cells. In the current study, we observed a significant association of integrin β1 with lipid rafts in laryngeal cancer cells using morphological methods and biochemical isolation of lipid raft fractions. We found that disruption of lipid rafts by MβCD did not influence the expression of integrin β1. These data were in line with the previous studies on the functional role of MβCD [14]. In addition, disruption of lipid rafts could regulate the invasion and radiosensitivity of laryngeal cancer cells. Based on our results, it is possible that the intact lipid rafts might serve as a signaling platform for integrin β1.

## Conclusions

In conclusion, our data provide strong evidence for the contribution of integrin β1 to the invasion and radioresistance of laryngeal cancer cells. Integrin β1 could interact with CD147 in laryngeal cancer cells. We also provide evidence that lipid rafts play important roles in integrin-β1-mediated malignant phenotypes. Therefore, integrin β1 has potential as a therapeutic target in prevention and treatment of laryngeal cancer.
